# Exploring cavity dynamics in biomolecular systems

**DOI:** 10.1186/1471-2105-14-S19-S5

**Published:** 2013-11-12

**Authors:** Norbert Lindow, Daniel Baum, Ana-Nicoleta Bondar, Hans-Christian Hege

**Affiliations:** 1 Department of Visualization and Data Analysis, Zuse Institute Berlin, Takustr. 7, 14195 Berlin-Dahlem, Germany; 2 Theoretical Molecular Biophysics, Department of Physics, Freie Universität Berlin, Arnimallee 14, 14195 Berlin-Dahlem, Germany

## Abstract

**Background:**

The internal cavities of proteins are dynamic structures and their dynamics may be associated with conformational changes which are required for the functioning of the protein. In order to study the dynamics of these internal protein cavities, appropriate tools are required that allow rapid identification of the cavities as well as assessment of their time-dependent structures.

**Results:**

In this paper, we present such a tool and give results that illustrate the applicability for the analysis of molecular dynamics trajectories. Our algorithm consists of a pre-processing step where the structure of the cavity is computed from the Voronoi diagram of the van der Waals spheres based on coordinate sets from the molecular dynamics trajectory. The pre-processing step is followed by an interactive stage, where the user can compute, select and visualize the dynamic cavities. Importantly, the tool we discuss here allows the user to analyze the time-dependent changes of the components of the cavity structure. An overview of the cavity dynamics is derived by rendering the dynamic cavities in a single image that gives the cavity surface colored according to its time-dependent dynamics.

**Conclusion:**

The Voronoi-based approach used here enables the user to perform accurate computations of the geometry of the internal cavities in biomolecules. For the first time, it is possible to compute dynamic molecular paths that have a user-defined minimum constriction size. To illustrate the usefulness of the tool for understanding protein dynamics, we probe the dynamic structure of internal cavities in the bacteriorhodopsin proton pump.

## Background

Proteins are dynamic molecules and their dynamics are coupled to changes of the internal cavities of the protein. We are particularly interested in understanding how conformational changes that accompany the reaction path of molecular transporters couple to changes in the number, shape, and volume of internal cavities that may host water molecules. Classical all-atom molecular dynamics simulations can provide detailed molecular movies - that is, *trajectories *- of the protein motions for a certain period of time. Typically, this period lies in the range of tens or hundreds of nanoseconds. Such a trajectory contains the time-dependent Cartesian coordinates of each atom in the simulation system. The number of coordinate sets in the trajectory depends on the length of the simulation and on the step size used for solving numerically the classical mechanical equations that describe the interactions between the various atoms of the simulation system [1].

We have developed a tool that allows the user to interactively explore the dynamics of cavities in biomolecules. This tool is based on the computation of Voronoi diagrams of spheres from which geometrical molecular paths are derived for a single configuration [2]. Each molecular path is associated with a cavity that can be represented as the union of a set of empty spheres with maximal radius positioned along the path. In our previous work [3], we extended the method for static molecules to the computation of dynamic cavities in molecular dynamics trajectories. In the current paper, we further extend the analysis of the cavity dynamics. The main improvements we present here are as follows: (1) Interactive computation and visualization of cavity volumes. (2) Computation of the intersection volumes of cavities to improve the visual tracing. (3) Improved tracking of single cavities and the visualization of tracked cavities using volume rendering. For the purposes of the current work, we do not distinguish between cavities and channels (extended cavities that connect at least two sides of the protein). We chose the bacteriorhodopsin proton pump as model system to test and illustrate the usefulness of our method. This choice is motivated by observations that the pumping cycle in bacteriorhodopsin is coupled to changes in the location of internal water molecules (see, for example, [4,5]). Because vectorial proton pumping requires tight control of the accessibility to the bulk of the internal proton donor and acceptor groups, understanding the dynamics of internal cavities large enough to host water molecules is an important step towards analyzing the proton-coupled protein and water dynamics of the pump.

**Figure 1:**
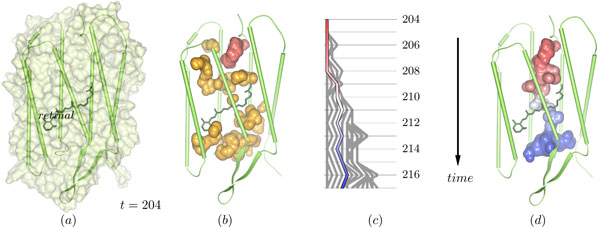
**The cavity structure in a bacteriorhodopsin monomer**. (a) The monomer at time step 204 is depicted by its surface and secondary structure cartoons. The small structure in the middle of the monomer, shown by its bonds, is the retinal, which is covalently bound to Lys216. (b) Depicted are all cavities that were found in time step 204. From the red cavity, the tracing is started (see panels (c) and (d)). (c) Split and merge graph showing the path tracing started at the red cavity in (b). (d) The selected path in (c) started at the red cavity leads to the dynamic molecular path depicted in this image. The path connects the cytoplasmic (top) and the extracellular side (bottom).

For the analysis presented here, we used the last 20 ns of an approximately 90 ns molecular dynamics trajectory of a bacteriorhodopsin trimer in a hydrated lipid membrane patch. We took 2000 equally-spaced coordinate sets of one monomer (see Figure 1). Details of how the molecular dynamics simulations were performed are given in our previous work [3]. Note that throughout the current report, we used a molecular dynamics run that differs from the previous one [3] in the starting geometry of an inter-helical hydrogen bond.

### Related work

Numerous approaches for computing cavities in biomolecules have been reported, and a thorough overview of these approaches is given in the works of Lindow et al. [2,3]. One of the first approaches is HOLE [6], which uses simulated annealing to place spheres inside cavities, whereas HOLLOW [7] is based on a grid data structure. Recently, a new method was proposed that is based on sampling points around a molecule together with a graph-based approach to identify cavities [8]. The approach comes with a brushing and linking visualization system.

Other methods for computing cavities are based on geometric molecular paths [9-12]. These approaches are similar to the one used in this paper. Briefly, geometric paths are either computed using a grid data structure on which Dijkstra's shortest path algorithm is run [9], or by applying the concept of the Voronoi diagram, or the Delaunay triangulation. Using a grid data structure or the classical Voronoi diagram [10-12] gives only an approximation of the geometric paths. In contrast, our approach of using the Voronoi diagram of the van der Waals spheres of the atoms results in an exact determination of the geometric paths [2].

A key aspect of our method is that we can analyze the dynamics of internal cavities. There exist two other approaches for analyzing cavities in time-dependent data [13,14]. One of these approaches [13] is based on using residence probabilities as means to include the dynamics of the molecule. In the second work [14], one starts with a selection of an internal cavity, which is represented by voxels. These voxels are then interactively traced and analyzed over the trajectory until outside of the molecule is reached. The key limitation of these two previous approaches is the dependence on the resolution of a grid data structure.

This limitation is overcome in our Voronoi-based method, since for the representation of the time-dependent molecular paths, we use both an analytic description of the geometry of the paths and their spatial extension.

## Methods

The computation of dynamic molecular paths consists of two steps. In the first step, the pre-processing step, we compute the static molecular paths for each time step separately. For this purpose, we apply the Voronoi-based path computation techniques [2], which we summarize first. Afterwards, we describe the computation of the cavity volume and the intersection volume of two cavities before we describe the second stage, in which paths are interactively traced over time.

### Definitions

Before we compute molecular paths and cavities, we define them mathematically and describe their relationship to each other. Our definition of a molecular path is based on the geometry of the empty space around the atoms. Throughout this paper, a *static molecular path *is a 3-dimensional continuous curve inside the molecule whose distance to the van der Waals surface is maximal and does not fall below a given minimal value *r_p _*- the radius of a sphere called *probe sphere*. From the mathematical viewpoint, a static molecular path is a subset of the topological skeleton of the distance function of the van der Waals spheres. It consists of maxima, index-2-saddles, and the connecting separatrices. All static molecular paths of a molecule together represent a graph. We call the connected components of this graph *path components*. The space around a path component where the probe sphere can be placed without intersecting the molecule is called *cavity*.

Based on the definition above, we can now define a *dynamic molecular path *as a collection of static path components that are connected over time along the trajectory. To form a dynamic path, the cavities of the static path components of consecutive time steps must have sufficient geometrical overlap. This means, that the probe sphere must be able to move from the cavity of the static path of one time step into the cavity of the static path of the following time step without leaving the intersection of the two cavities.

### Computation of static paths

**Figure 2:**
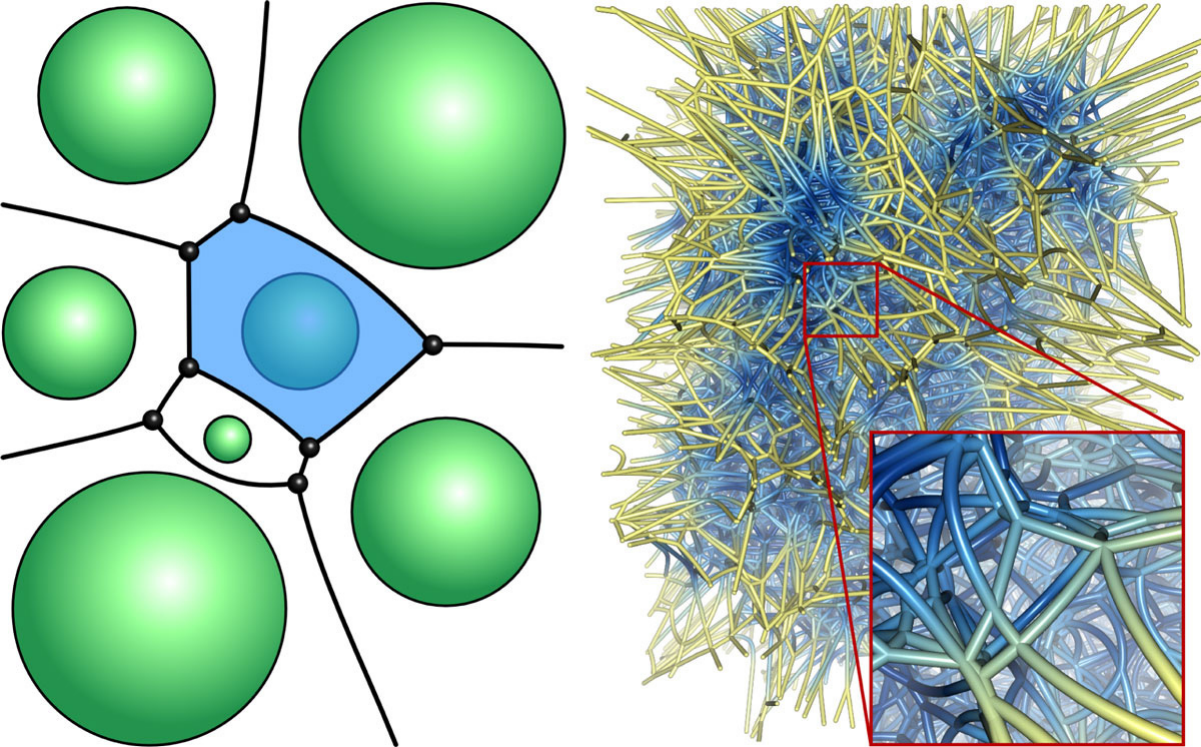
**Voronoi diagram**. In the left image, a two-dimensional illustration of a Voronoi diagram of spheres is shown. A single Voronoi region has been highlighted in blue. On the right side, the Voronoi diagram of spheres for a selected time step of the bacteriorhodopsin trajectory is shown. The Voronoi edges and vertices were cut with the bounding box of the protein, and the edges have been colored with respect to the distances to the van der Waals surface of the protein. Here, blue corresponds to small distances and yellow to large distances.

The complete topology of the distance function of the van der Waals spheres of a molecule is described by the Voronoi diagram of the van der Waals spheres. Consider a molecule with *n *atoms whose positions are $p_{i}\in {\mathbb{R}}^{3}$ and whose van der Waals radii are $r_{i}\in \mathbb{R}$, with *i *= 1, ..., *n*. The three-dimensional Voronoi diagram of spheres decomposes ${\mathbb{R}}^{3}$ into regions, facets, edges, and vertices. For each atom *i*, a Voronoi region *V_i _*is defined as the set of all points, whose distance to the corresponding atom sphere is equal or smaller than to any other atom sphere. Mathematically it is given by *V_i _*= {*p *| ||*p *- *p_i_*|| - *r_i _*≤ ||*p *- *p_j_*|| - *r_j_*, *i *≠ *j*}. The facets, edges, and vertices are defined as two-, one-, and zero-dimensional non-empty intersections of the Voronoi regions. A Voronoi diagram is called non-degenerated, if the input spheres lie in general positions, so that each facet is defined by the intersection of exactly 2 regions, each edge by the intersection of exactly 3 regions, and each vertex by the intersection of exactly 4 regions. Most algorithms can compute only non-degenerated Voronoi diagrams. In order to avoid degenerated cases, each atom position is perturbed by an individual random vector whose length is small enough that the resulting error is irrelevant for the structure analysis. An example of a two-dimensional Voronoi diagram is illustrated in Figure 2, left. The three-dimensional Voronoi diagram of the atom spheres of one time step of bacteriorhodopsin is shown in Figure 2, right.

Based on the definition of a Voronoi diagram, the graph given by the Voronoi vertices and edges describes all paths in a molecule, whose distance to the atom spheres becomes maximal. This means, all maxima, index-2-saddles, and the connecting separatrices are described by the Voronoi vertices and edges. For this reason, during the Voronoi diagram computation, we focus only on this graph. The first algorithm to compute a Voronoi graph for three-dimensional spheres was presented by Kim et al. [15]. A revised and optimized version of this algorithm is described in our previous work [2].

After the computation of the Voronoi graph, vertices and edges that are not part of static molecular paths need to be filtered, because these elements are either too close to the atoms or lie outside the domain of the molecule. For the filtering of paths outside the domain of the molecule, we use an ambient occlusion approach. The more a Voronoi vertex is surrounded by atoms, the less ambient light is received at its position. We geometrically approximate the ambient light for each vertex and remove all vertices that receive more light than a user-defined threshold. To ensure that the probe sphere never intersects the atoms along the paths, we further filter all vertices and edges whose distance to an atom sphere is smaller than the radius *r_p _*of the selected probe sphere. Implementation details for this filtering are given in our previous work [2].

### Cavity analysis

After filtering, the remaining graph of Voronoi vertices and edges contains all static molecular paths accessible to the selected probe sphere. Remember that each Voronoi vertex defines an empty sphere tangent to four atom spheres whose Voronoi regions created the vertex. Furthermore, each point on a Voronoi edge defines an empty sphere tangent to the three atoms whose Voronoi regions created the edge. For the further analysis and visualization, we approximate each cavity corresponding to a path component by the union of the empty spheres at the Voronoi vertices and a set of empty spheres on the Voronoi edges. The quality of this approximation naturally depends on the sampling density along the edges. The empty spheres along the Voronoi edges are placed such that the largest circle inside the intersection of neighboring spheres is at least *r_p_*. With this criterion, a probe sphere is guaranteed to be able to move along all the paths of a path component without colliding with the surface of the cavity approximated by the set of empty spheres. In the following, we use the term cavity also when we speak about the approximation described in this section.

The analysis of the dynamics of cavities involves computing the volumes of single cavities or the intersection volume of two cavities. A correct computation of the volume of a set of spheres, where the spheres can intersect each other, was described, for example, by Gibson et al. [16] and Petitjean et al. [17]. Depending on the input spheres, these algorithms are often complex, difficult to implement, and numerically unstable. We, therefore, compute an approximation of the volume using a discretization of ${\mathbb{R}}^{3}$ into small cubes with side length *a*. For this discretization, we define the volume of a cavity as the sum of the volumes of all cubes whose centers lie inside at least one sphere of the cavity. If *a *approaches 0, the approximated volume converges to the correct volume of the cavity. The intersection volume of two cavities can be approximated in a similar way. It is the sum of the volumes of all cubes whose centers lie inside at least one sphere of each cavity. Using this discretization, we implemented a highly parallel algorithm in OpenCL [18] which computes the volumes on the GPU in real time. In the following, we first describe the algorithm that approximates the volume of a single cavity. We then extend the method to compute the intersection volume of two cavities.

**Figure 3:**
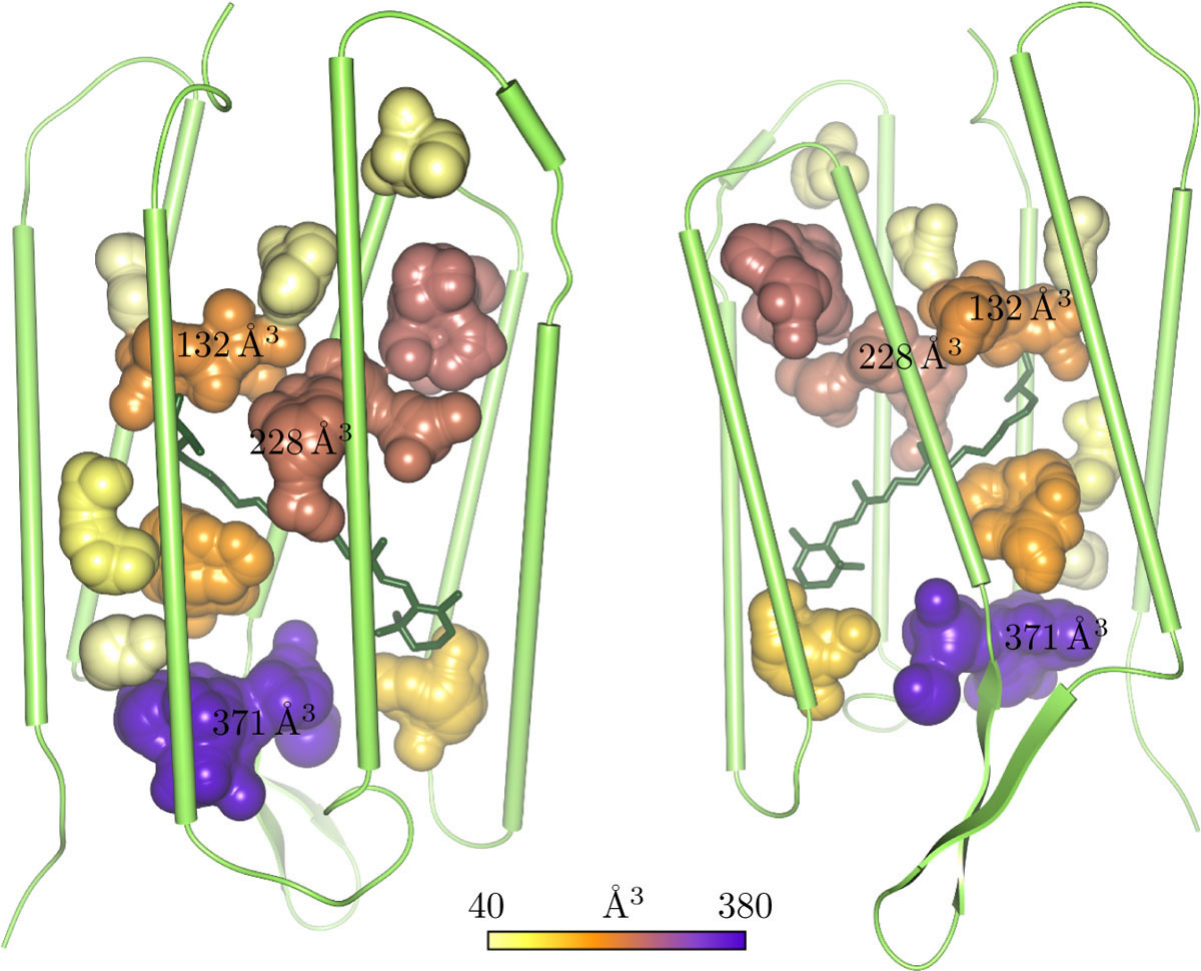
**Cavity volumes**. These images show two views of the same time step of bacteriorhodopsin with their cavities colored according to their volumes.

**Figure 4:**
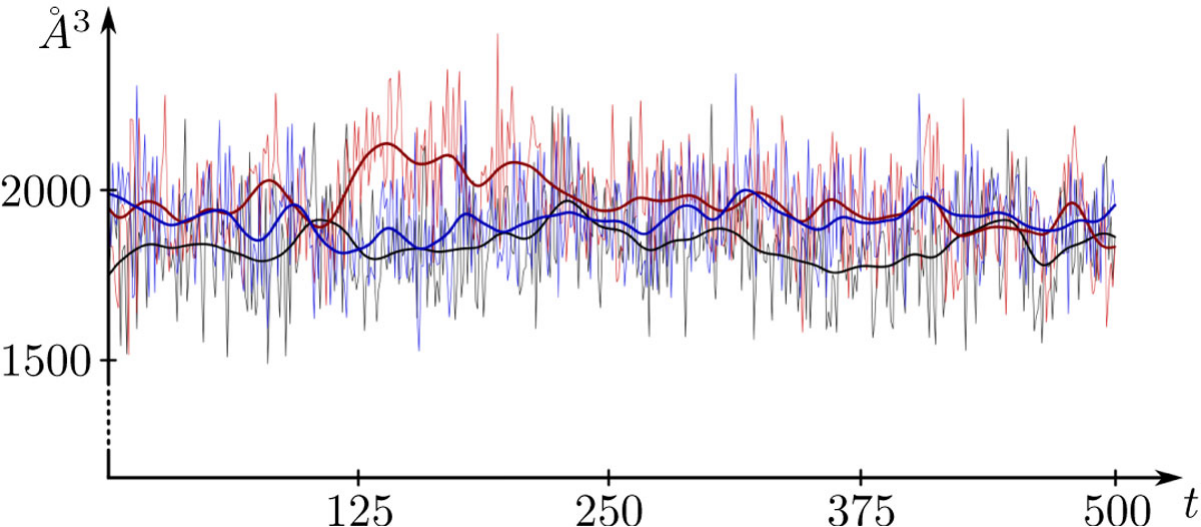
**Cavity volumes of the 3 monomers of bacteriorhodopsin**. In this plot, the accumulative volume of all cavities of the three monomers (red, blue, black) of bacteriorhodopsin are shown over 500 time steps. The smooth lines represent the volume plots smoothed with a Gaussian filter to show the tendency of the volume variations.

The first step is to compute the axis-aligned bounding box of the set of spheres representing the cavity. Then, we regularly sample the minimal x-y-facet of the bounding box w.r.t. the user-selected side length *a *of the cube. For each sample, we create an independent thread that computes the volume along the z-direction. For this, we regularly sample the z-direction again with *a *from the minimal to the maximal z-value of the bounding box. For each sample, we test if it lies inside a sphere or not. In order to perform this in a fast way, we use a 3-dimensional grid data structure to store the spheres. The grid stores a sphere inside a cell if the sphere intersects the cell or completely lies inside it. In this way, for each sample along the z-direction, we detect the corresponding grid cell. Then we perform the distance test of the sample with all spheres inside the cell. Because the x- and y-coordinates of the grid cells are constant within a thread, the computation of the cell for each sample is greatly simplified. In Figure 3, two images of the same time step are shown, in which the cavities are colored according to their volumes. Figure 4 shows plots of the accumulative volume of all cavities for each of the three monomers of bacteriorhodopsin.

To compute the intersection volume of two cavities, we combine the two sets of spheres into a single one. The first *n *spheres represent all spheres of the first cavity followed by the spheres of the second cavity. The rest of the algorithm is similar to the one described above. We only have to modify the condition during the distance tests such that the sample must be inside at least two spheres where one sphere has an index smaller than or equal to *n *and the other has an index larger than *n*. The performance can be further improved by reducing the number of samples. Instead of the bounding box of the combined set of spheres, we can take the intersection of the bounding boxes of both cavities, which again is an axis-aligned bounding box. Then, we reduce the grid according to this bounding box and ignore all spheres that do not intersect the box.

### Tracing of dynamic paths and cavities

**Figure 5:**
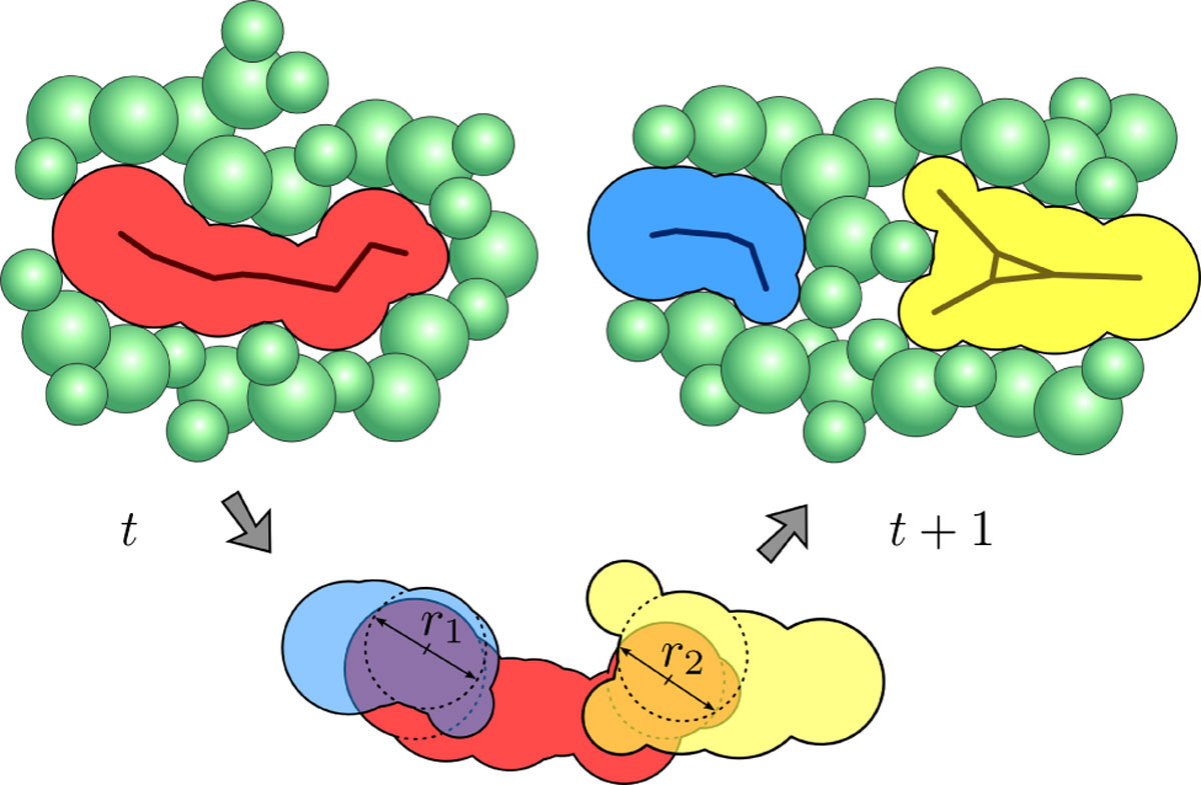
**Illustration of the path tracing algorithm in 2D**. The red component of time step t intersects with the blue and yellow components of time step t + 1. The maximal radii of these intersections are r1 and r2, respectively, depicted by the arrows. Since both radii are larger than the user-defined minimal radius of the intersection sphere, a split event is detected by the tracing algorithm.

The time evolution of path components can be assessed interactively once all static paths have been computed. Therefore, the description of a cavity is again given by the empty spheres of the corresponding path component. The tracing is steered by a manual selection of one or more start cavities of interest in an arbitrary time step. The selected cavities are traced over time while the user proceeds to the next time step. To do so, the assignment between two cavities of consecutive time steps is computed by the largest intersection circle of an empty sphere of the first cavity and an empty sphere of the second cavity. If the radius of this largest circle is greater than a user-defined minimal intersection radius *r_ic_*, the two cavities correspond to each other, otherwise they do not (see Figure 5). In principal, *r_ic _*should be equal or similar to *r_p_*, which is the radius of the probe sphere used for the filtering of the paths. In detail a tracing step is performed in the following way.

For each empty sphere *s *of each cavity in time step *t*, we detect all empty spheres of all cavities in time step *t *+ 1 that intersect *s *with an intersection circle larger than the user-defined circle *r_ic_*. For all pairs of spheres that fulfill this condition, the corresponding cavities are mapped onto each other. In our case, the intersection circle of two spheres is defined as the largest circle inside the intersection volume of both spheres.

In order to illustrate the assignment of the cavities during the tracing, an undirected graph, called 'time graph', is created. Each vertex in this graph represents a cavity at a certain time step. Two vertices of consecutive time steps share the same edge if the corresponding cavities are mapped onto each other during the tracing. In this way, events like cavity splits and merges are represented by the graph.

**Figure 6:**
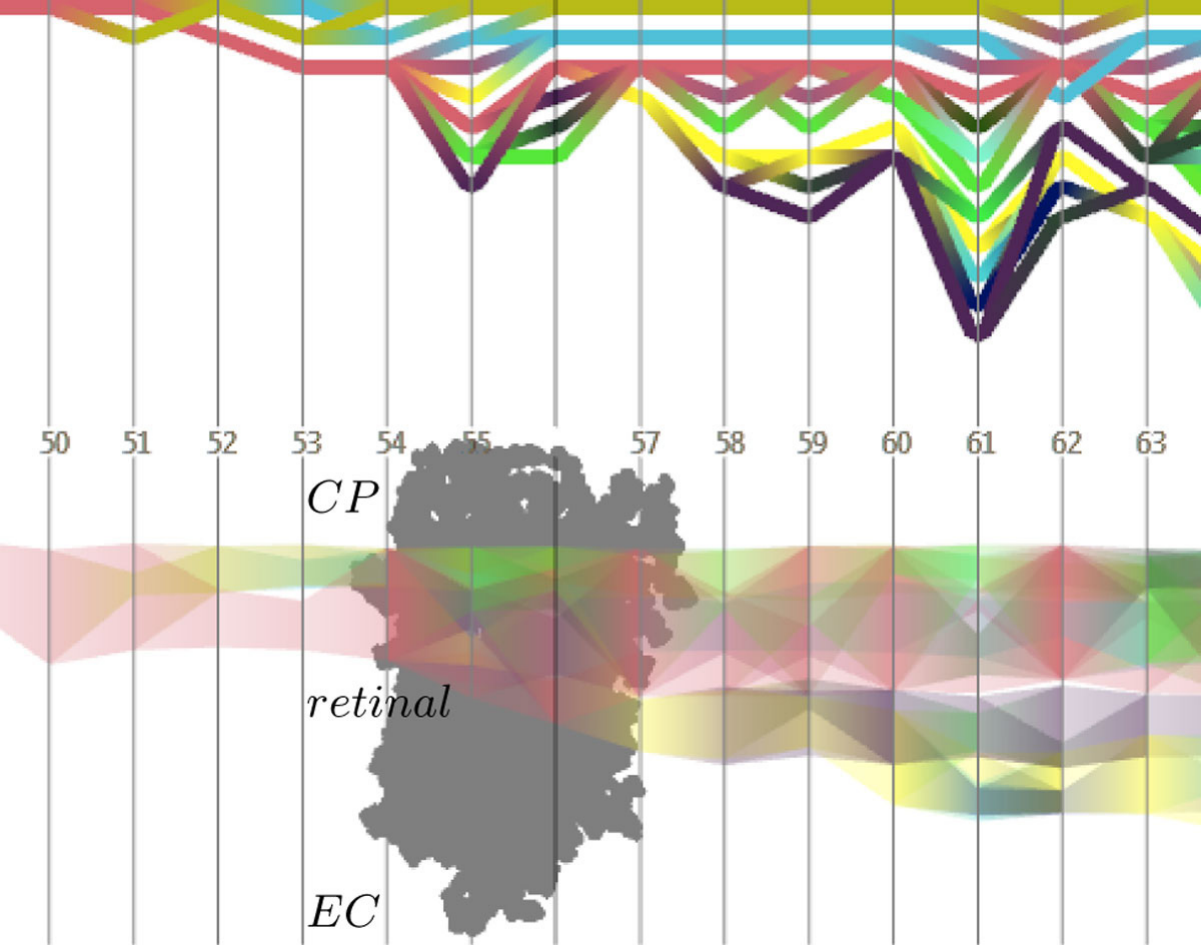
**Path timeline visualizations**. The split and merge graph (top) shows topological events of the time-varying cavity structure. The evolution graph (bottom), on the other hand, depicts the position and size of the cavities along a user-defined direction. The time steps of the trajectory are given by the displayed numbers. At time step 55, a temporary connection between the cytoplasmic (CP) and the extracellular (EC) cavities occurs, which, however, does not represent the formation of a water-filled channel. To ease the understanding of the displayed events, a projection of the molecule is displayed in the evolution graph.

In addition to the graph creation, we compute identification numbers for the traced cavities. Each identification number is related to a specific color and allows the user to easily visually trace single cavities over time (see Figure 6). To represent the assignments of cavities, the identification numbers are computed by the following iterative approach. Assuming that we have already computed the identification numbers for all cavities in time step *t*, we now determine the numbers in *t *+ 1. Therefore, we compute a matching in the bipartite subgraph, which contains the cavities of *t *and *t *+ 1 and the edges between them. This matching represents the best mappings of the tracing, so if cavity *c_t _*is matched to cavity *c*_*t*+1_, then *c*_*t*+1 _gets the same identification number as *c_t_*. All unmatched components of *t *+ 1 are splits and all unmatched components of *t *are not traced to *t *+ 1 or merge into one or more components of *t *+ 1. The main problem is the definition of a matching criteria that represents the best tracing correlations. This is mathematically difficult and might be ambiguous. Hence, we propose the following heuristic.

For each edge in the subgraph, we compute the intersection volume of the corresponding cavities, as described above. Then we sort the edges according to the intersection volumes, starting with the largest one. Let *c_t _*and *c*_*t*+1 _be the cavities of the first edge in the list. If one of the two components is already matched, we ignore this edge and continue with the next in the list. Otherwise, we match *c_t _*with *c*_*t*+1_ . We repeat this procedure until all edges in the list are processed. In the last step, we set the identification numbers according to the matching. For all unmatched components in *t *+ 1, we set new identification numbers so that finally each component in a time step gets a unique number. For the initial time step we just number the cavities consecutively.

**Figure 7:**
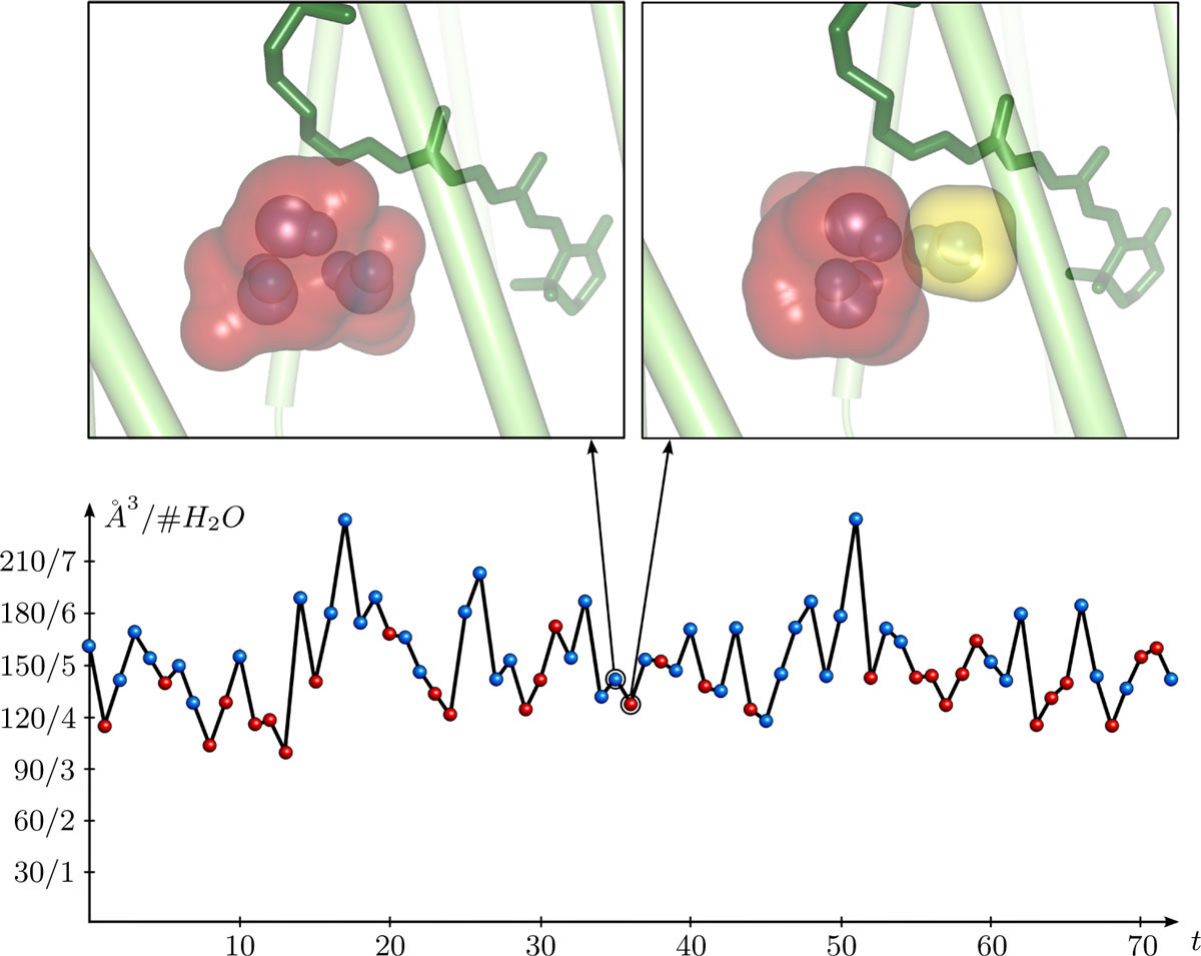
**Single cavity tracing**. Bottom: Volume plot of a single cavity traced over 72 time steps. The point color highlights whether two (red) or three (blue) water molecules are contained in the cavity. The labeling of the y-axis gives the volume numbers as well as the corresponding water molecules that would approximately fit into the volume, where the volume of a water molecule is approximated by 30 Å^3^. Top: The two images on top of the graph show the traced cavity (red) for two selected time steps together with the contained water molecules. In the right image, it can be seen that the traced cavity has split. A second, smaller cavity (yellow) contains the third water molecule.

A further feature of our algorithm is the detection and removal of dead ends. Dead ends are cavities that cannot be mapped to a cavity in the following time step during the tracing. These cavities are identified, removed and traced back in time until a splitting in the time graph is found. This allows the user to focus on stable cavities and to reduce visual clutter. Another option in our tracing system is to forbid splits. For this, we keep only the matched cavities of *t *+ 1 and remove all unmatched ones. This allows the user to explicitly follow single cavities and analyze their locations and dimensions. In Figure 7, we have plotted the absolute volume of a single cavity that was traced over time. The tracing of single cavities together with the volume computation also allow us to compare the volume of the cavity with the actual number of water molecules contained in the cavity (Figure 7).

### Visualization system

Based on the definitions and descriptions given in previous sections, we developed a visualization system for interactive path exploration. This system offers all common molecular representations, such as ball-and-stick, molecular surfaces, and secondary structure. The surface representations include the van der Waals surface, the solvent accessible surface (SAS), the solvent excluded surface (SES), and the molecular skin surface (MSS). Since the surface computation and rendering techniques are interactive [19], they are directly suitable for dynamic molecular data. Onto all molecular representations, attributes can be mapped using pseudo-coloring. These attributes can represent properties of atoms, residues and functional groups. Furthermore, filters can be applied to hide parts of the molecule that are of less interest.

For visualizing molecular paths, we render the filtered Voronoi diagram, where each vertex is depicted by a small sphere, and each edge that connects two vertices is depicted by a cylinder. The rendering of a cavity is done using the skin surface [19,20] of the empty spheres approximating the cavity. We use the identification number to color the cavities. This allows the user to easily trace cavities when going from one time step to the next [3].

### Timeline visualizations

While the 3-dimensional visualization of cavities provides a good representation of their size and location, it does not necessarily allow efficient detection of splitting and merging events that can occur during the trajectory. For this reason, we developed two different timeline visualizations. These 2-dimensional graph representations show topological and geometrical changes in a user-defined time range (see Figure 6). Note, that the range of time can be changed interactively.

#### Split and merge graph

The first graph representation shows topological events like splits and merges. Therefore, each traced cavity is visualized as a polyline from left to right, representing the direction of time. If during the tracing a cavity splits into two or more cavities, the corresponding polyline also splits. Accordingly, polylines merge if the corresponding cavities merge. Due to the splits and merges, intersections of polylines can occur over time. We could possibly reduce the number of intersections using an optimized graph layout. Currently, however, the cavities are simply rendered from top to bottom. To keep consistency between the 3-dimensional visualization and the timeline graph, the same colors are used for both representations. Additional information, like the size of a cavity, can be encoded by the line thickness. Figure 6, top, shows an example of a split and merge graph.

#### Evolution graph

The second timeline visualization shows the evolution of the traced cavities along a user-defined direction. Especially for membrane molecules, whose main path direction is often along the membrane normal, it is interesting to analyze the geometrical evolution of the cavities over time. Similar to the split and merge graph, each cavity is depicted as a polyline from left to right. The main difference is that the position and the thickness of a line depends on the position and expansion of the corresponding cavity along the selected direction. Using alpha blending, we avoid occlusion of lines. In addition, we add an orthogonal projection of the molecule to the background of the graph. This helps to identify the location of the cavities and to stay connected with the 3-dimensional view. One can see an example of an evolution graph of bacteriorhodopsin in Figure 6, bottom. A single cavity that was traced creates a dynamic channel from the cytoplasm side (top) to the extracellular side (bottom). During the first time steps, only cavities above the retinal get interconnected. Then, at time step 55, the cavities on the cytoplasmic side of the retinal connect with cavities on the extracellular side. We would like to stress that within our current analysis tool, the connectivity across the retinal Schiff base region is a pure geometrical construct: it does not necessarily imply that a physically stable channel forms through the retinal region.

### Visualization of cavity dynamics

Instead of only animating the traced cavities and the molecular structures over time, we also enable the user to visualize the dynamics of a cavity as compact representation in a single image. Let us recall that a dynamic path is a union of static path components that are connected over time. The user can extract an arbitrary dynamic path by selecting a path in the split and merge graph. To assist the selection, the evolution graph can be used for an overview of the progression of penetration. Once a time region of particular interest has been identified, the user can select a cavity in the split and merge graph. The end of the dynamic path is given by a further selection of a cavity in a subsequent time step. Then the path between these two cavities is computed by a modified depth first search. A different path can be achieved by adding further intermediate selections.

**Figure 8:**
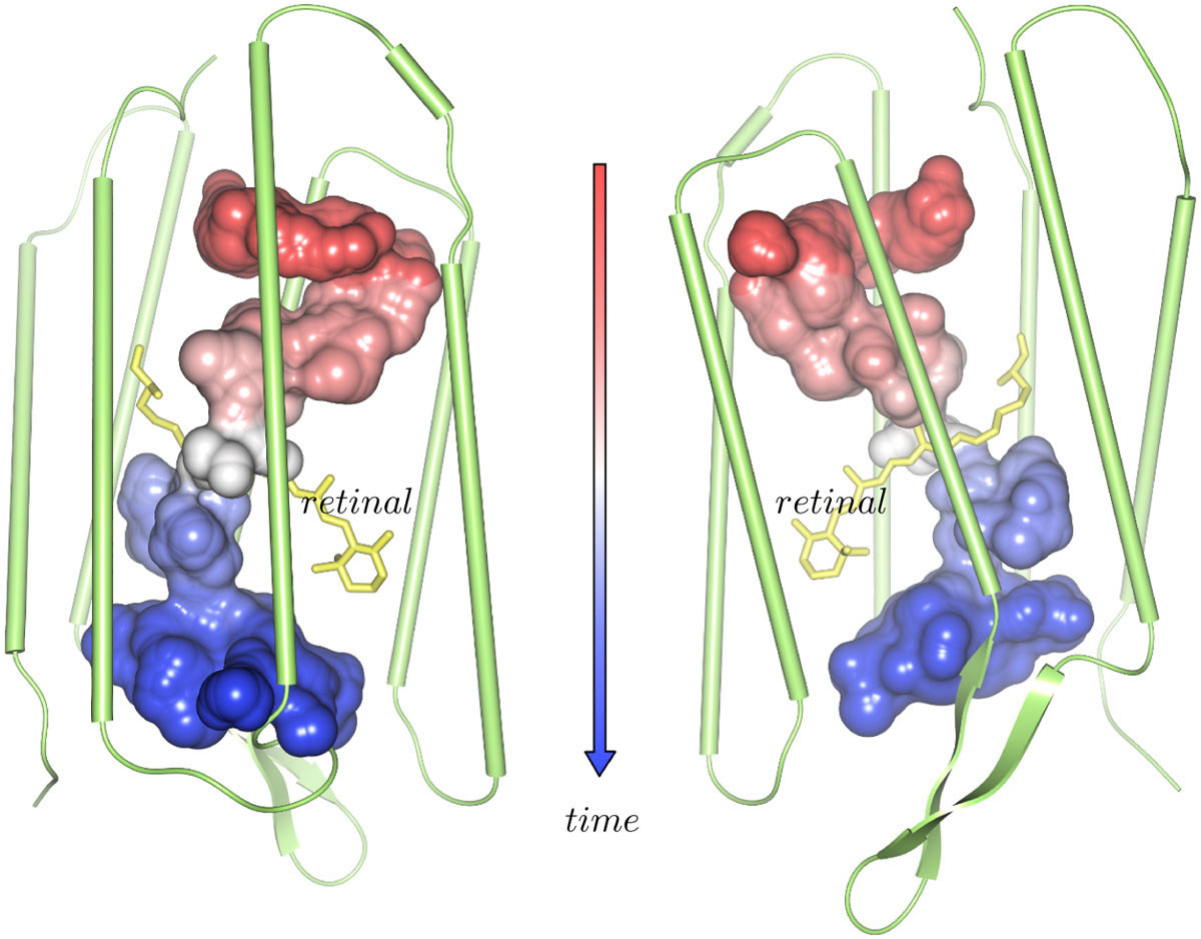
**Dynamic molecular cavity**. To obtain the displayed surface of the dynamic cavity, all empty spheres all cavities that are part of the selected traced path are displayed using the skin surface. The surface is colored according to the time, where red corresponds to an early time and blue to a later one. Between starting and end point, the time is linearly interpolated and mapped to the color map.

The dynamic cavity corresponding to the selected path can be rendered efficiently using again the skin surface approach. Therefore, we compute the skin surface of all empty spheres of all cavities belonging to the dynamic path. Thus, we get a static representation of a dynamic process. To still keep track of the path dynamics, we add a color-coding according to the time evolution of the cavities (see example in Figure 8).

### Residence probability visualization

**Figure 9:**
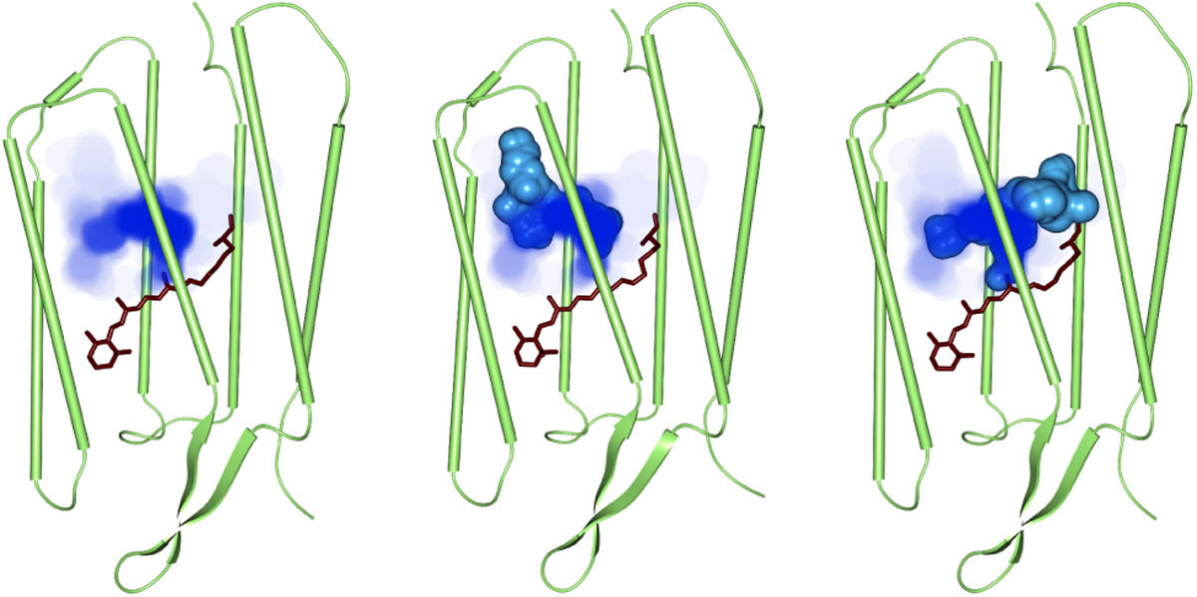
**Residence probability of a single dynamic cavity in bacteriorhodopsin**. The residence probability of the traced dynamic cavity is displayed using the maximum intensity projection. In the middle and right images, the static cavities together with the dynamic cavity at two different time steps are shown as skin surface.

Visualizing the skin surface of a dynamic cavity is suitable to analyze its maximal dimension and dynamic progress, but sometimes the user is more interested in the residence probability instead of a hard boundary. This could be the case, for example, when a single cavity is traced that has a stable core. For the time interval of the dynamic cavity, we approximate its residence probability by the proportion of time in which a point is inside the cavity. In practice, this is done by regularly sampling ${\mathbb{R}}^{3}$ inside the axis aligned bounding box of the dynamic cavity. Such a sampling can be visualized using volume rendering or iso-surfaces. For volume rendering, we suggest to use a maximum intensity projection, which highlights fixed cores. In Figure 9, the maximum intensity projection (MIP) of the residence probability of a single dynamic cavity is shown together with two corresponding static cavities of two time steps. The stable core can clearly be distinguished from the more unstable cavity regions. Similar techniques are used, for example, in uncertainty visualization [21,22].

In order to derive an overview of the cavities throughout the entire trajectory, the user can compute the residence probability for all cavities over the whole time interval of the trajectory (see our previous work [3]).

## Results

The algorithms described here require the setting of parameters for the filtering and visualization of molecular paths and cavities as well as for the computation of the cavity volumes.

The filtering of molecular paths requires two parameters. The first parameter is the radius of a probe sphere that approximates the accessibility of a solvent or substrate. If the user is interested in cavities large enough to host a water molecule, a starting value for the radius of the probe could be 1.4 Å. We used this value for both, the path filtering and the tracing of cavities. The second parameter for the path filtering is the threshold used to remove paths that are outside the molecule [2]. Here we used a threshold of 15%. Visualization of the cavities requires a parameter for the skin surface, called shrink factor. Too small values of the shrink factor can lead to intersections between the skin surface and the van der Waals surface. Our tests indicate that such intersections can be avoided by using values of at least 0.7. Moreover, the error between the skin surface and the correct surface of the cavities becomes very small. The last parameter is used for the computation of the cavity volumes. Here, we used a cube length of *a *= 0.2 Å.

### Performance

Detailed information on the performance for the computation and visualization of molecular paths have already been reported in our previous work [2]. Here we present the performance measures for the analysis of one monomer of bacteriorhodopsin (3624 atoms) using the 2000 equally-spaced coordinate snapshots from the last 20 ns of the molecular dynamics trajectory. For a single time step, the computation of all paths took approximately 1.3 s on an Intel Xenon 5650 with 2.67 GHz. Altogether, the pre-processing took 44 min.

The most expensive step for the visualization is the rendering of the solvent-excluded surface together with a clipping of the surface by cavities. To measure the graphics performance, we rendered the surface using an NVIDIA Geforce GTX 470 graphics card with a screen resolution of 1024 × 1024 and an average fill rate of 75%. Depending on the view direction and the time step, this visualization was done with 40 to 50 fps. The dynamic visualizations of consecutive time steps requires updating the molecular and the cavity surfaces; these dynamic visualizations gave lower frame rates of approximately 25 fps. In the examples given here for bacteriorhodopsin, the computation of the cavity volumes and of the intersection volumes of two cavities was fully interactive. Compared to an implementation that ran on a single CPU, we obtained a speed-up of approximately 50 times using OpenCL [18] on the GPU. Given the fast computation of intersection volumes and the use of 3-dimensional data structures, the time for the path tracing is negligible.

### Paths in the bacteriorhodopsin proton pump

To test and illustrate the usefulness of the tool for biomolecules, we used a segment of 20 ns from the end of an equilibrated trajectory of a bacteriorhodopsin trimer embedded in a hydrated lipid bilayer. The starting crystal structure [23] indicates coordinates for water molecules inside the protein. Of particular importance are water molecules close to the proton transfer groups. Consistent with previous molecular dynamics simulations [24] and reaction path computations [25], during the 90 ns of the molecular dynamics simulations, we did not observe events in which water molecules pass across the retinal Schiff base region.

The analysis of the cavities using the tools presented here allows us to identify cavities large enough to host water molecules and trace their dynamics. With the criterion used in the current tests, the cavity on the extracellular side of the retinal Schiff base appears to be large enough to contain four or even five water molecules, yet only three water molecules are found in the crystal structure and in the simulations (Figure 7). This is an interesting finding that clearly needs further investigation. The cavities on the extracellular side for two time steps together with the contained water molecules can be seen in the upper panels of Figure 7.

## Conclusion

Exploring the dynamics of internal cavities is of potential interest to the study of protein dynamics. Methods that allow interactive analysis of cavity dynamics are scarce. Such a method was presented recently in [26]. In this paper, we reported a versatile tool that brings the user the advantage of higher geometric accuracy, and advanced path and cavity visualization and analysis. To the best of our knowledge, the set of tools presented here and in our previous work [3] is the first that can be used to compute all possible geometric paths with a user-defined minimum constriction size for time-dependent data.

Taking advantage of the advanced interface, the user can apply our tool to identify cavities, trace their dynamics, and compute their time-dependent volumes. A global representation of the accessible regions in a biomolecule can be derived by combining the cavities computed at consecutive time steps into a single representation.

The residence probability of all cavities computed for a certain time range gives a good overview of cavity dynamics and could be used to identify cavities that could be subject to closer investigation. When computed for a single dynamic cavity selected by the user, the residence probability gives insight into the dynamics of a particular region of the protein. The residence probabilities can be easily visualized using isosurfaces or maximum intensity projection volume rendering. In the future, visualization of the cavity dynamics could be further enhanced by adding movement illustrations as suggested, for example, by Meyer-Spradow et al. [27].

The current implementation of the cavity analysis tools allows the user to exploit the timeline graphs to rapidly identify events where cavities split or merge. This task can become difficult in the case of graphs containing a large number of cavities. To circumvent this limitation, in the future we plan to optimize the layout of the split and merge graph.

Furthermore, we aim to refine our tool for the analysis of molecular transporters and protein complexes that catalyze proton transfer reactions. This will involve more stringent tests to map the cavities inside the protein to the actual water dynamics. A further improvement would be the use of the electrostatic protein environment for a more physical cavity detection. Of particular importance for the quality of the cavity analysis is the time resolution of the molecular dynamics trajectory. Since smooth dynamic cavities could be obtained with smaller step sizes than the used 10 ps, we suggest that for high-resolution cavity analysis one should generate trajectories with a smaller step size.

## Competing interests

The authors declare that they have no competing interests.

## Authors' contributions

ANB motivated this work and provided the data set as well as the biophysical background. NL, DB and HCH developed the main ideas underlying the presented methods. NL implemented the methods. All authors improved the work with many helpful discussions and contributed to writing the paper.

## Declarations

This publication was funded by the IEEE Symposium on Biological Data Visualization (BioVis) as a supplement of highlights.

The articles in this supplement have undergone the journal's standard peer review process for supplements. The Supplement Editors declare that they have no competing interests.

This article has been published as part of *BMC Bioinformatics *Volume 14 Supplement 19, 2013: Highlights from the 2nd IEEE Symposium on Biological Data Visualization. The full contents of the supplement are available online at http://www.biomedcentral.com/bmcbioinformatics/supplements/14/S19.
